# Supplementary material for: Dissecting the mechanism of atlastin-mediated homotypic membrane fusion at the single-molecule level
Source: Nat Commun. 2024 Mar 20;15:2488. doi: 10.1038/s41467-024-46919-z (PMC10954664; doi:10.1038/s41467-024-46919-z)
Supplement: Supplementary file 1 — Supplementary Information [file 41467_2024_46919_MOESM1_ESM.pdf]

Supplementary Information for

Dissecting the mechanism of atlastin-mediated homotypic membrane fusion at the single-molecule level

Lijun Shi[1,7], Chenguang Yang[2,3,7], Mingyuan Zhang[4], Kangning Li[1], Keying Wang[4], Li Jiao[5], Ruming Liu[5], Yunyun Wang[1], Ming Li[2], Yong Wang[4,6,*], Lu Ma[2,*], Shuxin Hu[2,*], Xin Bian[1,*]

[1]State Key Laboratory of Medicinal Chemical Biology, College of Life Sciences, Frontiers Science Center for Cell Responses, Nankai University, Tianjin 300071, China;

[2]National Laboratory for Condensed Matter Physics, Institute of Physics, Chinese Academy of Sciences, Beijing 100190, China;

[3]University of Chinese Academy of Sciences, Beijing 100049, China;

[4]College of Life Sciences, Zhejiang University, Hangzhou 310027, China;

[5]College of Life Sciences, Nankai University, Tianjin 300071, China;

[6]The Provincial International Science and Technology Cooperation Base on Engineering Biology, International Campus of Zhejiang University, Haining, 314400, China;

[7]These authors contributed equally

[*]Correspondence: xin.bian@nankai.edu.cn;
hushuxin@iphy.ac.cn;
luma@iphy.ac.cn;
yongwang_isb@zju.edu.cn

This PDF file includes:
Supplementary Figures 1-15 and Supplementary Tables 1-7

```
hsATL1   MAK-------NRRDRNSWGGF-----------------SEKTYE--WSSEEEEPVKKAGPVQV--LIVKDDHSFELDETALNRILLSEAVRDKEVVAVSV   72
hsATL2   MAEGDEAARGQQPHQGLWRRRRTSDPSAAVNHVSSTTSLGENYEDDDLVNSDEVMKKPCPVQIV-LAHEDDHNFELDEEALEQILLQEHIRDLNIVVVSV   99
hsATL3   ----------------------MLSPQ----RVAA--------AASRGADDAMESSKPGPVQVV-LVQKDQHSFELDEKALASILLQDHIRDLDVVVVSV   65
dmATL    -------------------------------------------------------MGGSAVQ--VINASEEHTFVLNEDALSEVLMRDEVKDRFVCVVSV   43
xtATL1   MAR-------NRKERNSWGGF-----------------TDRNYD--WSSEEEEYEKKAHPVQV--LVVKDDHSFELDEAALNRILLSEAVRDKEVVAVSV   72
ceATL1   ---------------------METTPQNEHNE-------HQQQQHAGHVEDVLLPKEPQAVRVVEVVEDTDHSFELNTELLEKILLDPKVADKKVAVIGV   72
aaATL    -----------------------------------------------------MAETAKPVQ--VVETGEEHSFTLNEDALTEILLQENVRDRTVVVISV   45
                                                                *:   :    :*.* *:  *  :*:   : *  : .:.*

hsATL1   AGAFRKGKSFLMDFMLRYMYNQE------------SVDWVGDYNEPLTGFSWRGGSERETTGIQIWSEIFLINKPDGKKVAVLLMDTQGTFDSQSTLRDS   160
hsATL2   AGAFRKGKSFLLDFMLRYMYNKD------------SQSWIGGNNEPLTGFTWRGGCERETTGIQVWNEVFVIDRPNGTKVAVLLMDTQGAFDSQSTIKDC   187
hsATL3   AGAFRKGKSFILDFMLRYLYSQKES---------GHSNWLGDPEEPLTGFSWRGGSDPETTGIQIWSEVFTVEKPGGKKVAVVLMDTQGAFDSQSTVKDC   156
dmATL    AGAFRKGKSFLLDFFLRYMYSKYVHHD--------ATDWLGGESDPLEGFSWRGGSERDTTGILMWSDIFLHDYPNGDKIAIILLDTQGAFDSQSTVRDC   135
xtATL1   AGAFRKGKSFLMDFMLRYMYKTD------------TVDWLGDYNEPLSGFSWRGGSERETTGIQIWSEIFLVENPDGNKVAVLLMDTQGTFDSQSTLRDS   160
ceATL1   AGAYRKGKSFLLNFFLRYLTWRSKADKVMGEVELDNSQWMS-PNSPLSGFSWRGGSERDTNGILIWSEPFIMKDKNGEEIAVLLMDTQGAFDSQSTVKDC   171
aaATL    AGAFRKGKSFLLDFFLRYMYSKYVHNK-------SASEWLGDENEPLTGFSWRGGSERDTTGILMWSDIFLHETPSGEKYAIILMDTQGAFDSQSTVRDC   138
         ***:******:::*:***:                 .*:.  ..** **:****.: :*.** :*.: *  .  .* : *::*:****:******::*.

hsATL1   ATVFALSTMISSIQVYNLSQNVQEDDLQHLQLFTEYGRLAMEETFLKPFQSLIFLVRDWSFPYEFSYGADGGAKFLEKRLKVSGNQHEELQNVRKHIHSC   260
hsATL2   ATVFALSTMTSSVQVYNLSQNIQEDDLQHLQLFTEYGRLAMEEIYQKPFQTLMFLIRDWSYPYEHSYGLEGGKQFLEKRLQVKQNQHEELQNVRKHIHNC   287
hsATL3   ATIFALSTMTSSVQIYNLSQNIQEDDLQQLQLFTEYGRLAMDEIFQKPFQTLMFLVRDWSFPYEYSYGLQGGMAFLDKRLQVKEHQHEEIQNVRNHIHSC   256
dmATL    ATVFALSTMLSSVQIYNLSQNIQEDDLQHLQLFTEYGRLALADTGKKPFQRLQFLVRDWSFPYEAEYGALGGDKILKRRLEVSDKQHPELQSLRRHISSC   235
xtATL1   ATVFALSTMISSIQVYNLSQNVQEDDLQHLQLFTEYGRLAMEETFLKPFQSLIFLVRDWSFPYEFPYGSDGGNKFLEKRLKVSENQHEELQNVRKHIHSC   260
ceATL1   ATIFALSTMISSVQIYNLSQNIQEDDLQHLQLFTEYGRLALEDSASKPFQSLLFLVRDWSFPYEAEFGFQGGQRVLDRRLEVSEKQHAELQQLRQHIRSC   271
aaATL    ATVFALSTMLSSVQIYNLSQNIQEDDLQHLQLFTEYGRLALADSGKKPFQRLQFLVRDWSFPYEAEYGASGGDVILKRRLEVQDKQHPELQSLRRHITSC   238
         **:****** **:*:******:******:***********: :   **** * **:****:***  :*  **  .*.:**:*.  :** *:*.:*.** .*

hsATL1   FTNISCFLLPHPGLKVATNPNFDGKLKEIDDEFIKNLKILIPWLLSPESLDIKEINGNKITCRGLVEYFKAYIKIYQGEELPHPKSMLQATAEANNLAAV   360
hsATL2   FSNLGCFLLPHPGLKVATNPSFDGRLKDIDEDFKRELRNLVPLLLAPENLVEKEISGSKVTCRDLVEYFKAYIKIYQGEELPHPKSMLQATAEANNLAAV   387
hsATL3   FSDVTCFLLPHPGLQVATSPDFDGKLKDIAGEFKEQLQALIPYVLNPSKLMEKEINGSKVTCRGLLEYFKAYIKIYQGEDLPHPKSMLQATAEANNLAAA   356
dmATL    FTEVACFLMPHPGLNVATNPKFDGRLQDITPEFKSSLRSLVPMLLAPDNLVYKEISGQRVRARDLIQYFQSYMNIYKGNELPEPKSMLVATAEANHLTAV   335
xtATL1   FTNISCFLMPHPGLTVATNPNFDGKLKEIDEEFIKNLKVFIPWLLSPENLDVKEISGNRITCRGLVEYFKAYIKIYQGEELPHPKSMLQATAEANNLAAV   360
ceATL1   FEDIRCFLMPHPGLKVATNPNFDGKLVDIENEFQQQLGVMIPRLLDSHALVHKEINGQKMTCRELLEYFKAYMHIFRGQDLPEPKSMLMATAEANNLAAV   371
aaATL    FTEIACFLMPHPGLTVATNQKFDGRLADITPEFKQSLKELVPMLLSPANLIPKEINGQKVKARDLVQYFKSYMAIYKGNELPEPKSMLVATAEANNLTAV   338
         * :: ***:***** ***. .***:* :*  :*  .*  ::* :*    *  ***.*.:: .* *::**::*: *::*::**.***** ******:*:*.

hsATL1   ATAKDTYNKKMEEICGGDKPFLAPNDLQTKHLQLKEESVKLFRGVKKMGGEEFSRRYLQQLESEIDELYIQYIKHNDSKNIFHAART   447
hsATL2   AGARDTYCKSMEQVCGGDKPYIAPSDLERKHLDLKEVAIKQFRSVKKMGGDEFCRRYQDQLEAEIEETYANFIKHNDGKNIFYAART   474
hsATL3   ASAKDIYYNNMEEVCGGEKPYLSPDILEEKHCEFKQLALDHFKKTKKMGGKDFSFRYQQELEEEIKELYENFCKHNGSKNVFSTFRT   443
dmATL    AAAKELYGQLMEEVCGGTRPYLSTAHLQTEHLRVKDKALFQFAAKRKMGGEEFTEKFRKQLEDDLEEVFTNYQAHNESKNIFKAART   422
xtATL1   ATAKDLYNKNMEEVCGGDRPFLAPTDLQQRHQHLKEEAVKLFRGVKKMGGEEFSRRYLQQLENEIDELYVQYIKHNDSKNIFHAART   447
ceATL1   ASARAVYQREMEEVCGGDTPYMSTNELLEHHDRVKNIAIREFRNARKMGGVDFSMQFLERLESDLQESYENYLKVNNGKNLFKSMRT   458
aaATL    AAAKDIYTQLMEDVCGGNKPYLNTAHLDSEHNRIKEKALHQFSSKRKMGGEEFSEKYRERLEQDLDDSYVTFKAHNESKNIFKAART   425
         * *:  * . **::***  *::    *  .*  .*: ::  *   :**** :*  :: ..** ::.: :  :   * .**:* : **
```

Supplementary Fig. 1 Sequence alignment of N-terminal cytosolic regions of ATLs from various species. The N-terminal cytosolic regions of seven ATLs were aligned using the program Clustal Omega (https://www.ebi.ac.uk/Tools/msa/clustalo/). The positions of dye-labeled sites

T51C and K400C are marked by red boxes. The positions of disease-causing mutations S398Y and N440T and of structure-disrupting mutation H443P are marked by blue boxes. hs, *Homo sapiens*; dm, *Drosophila melanogaster*; xt, *Xenopus tropicalis*; ce, *Caenorhabditis elegans*; aa, *Aedes aegypti*.

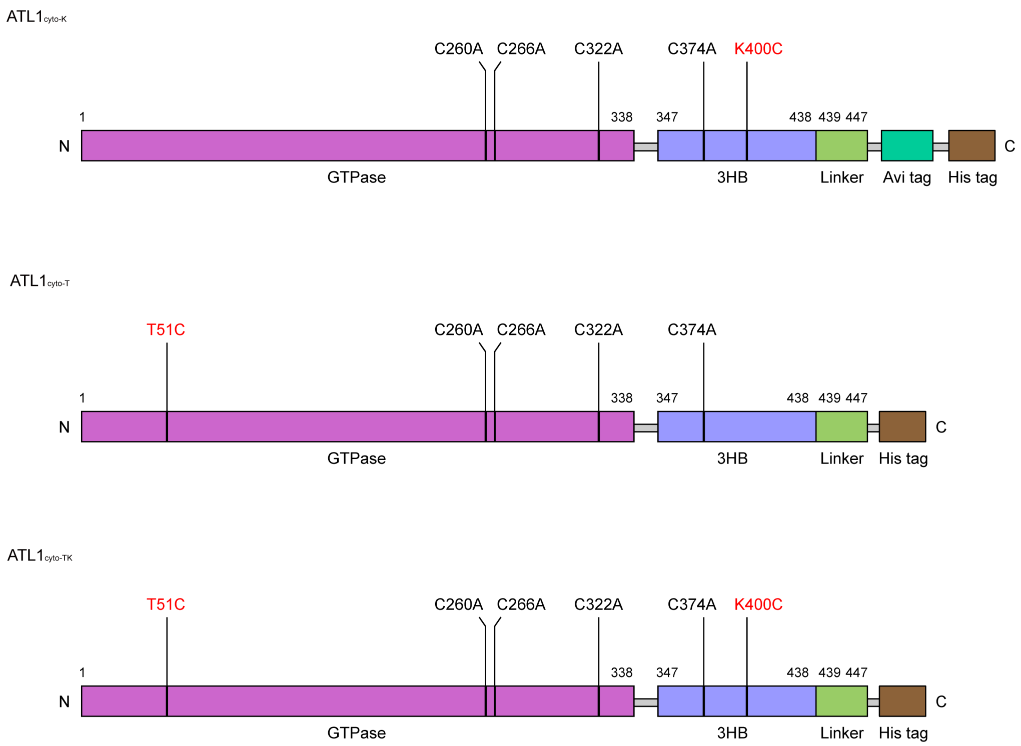

Supplementary Fig. 2 Domain structures of ATL1$_{\text{cyto-T}}$, ATL1$_{\text{cyto-K}}$, and ATL1$_{\text{cyto-TK}}$. The positions of the mutations are shown.

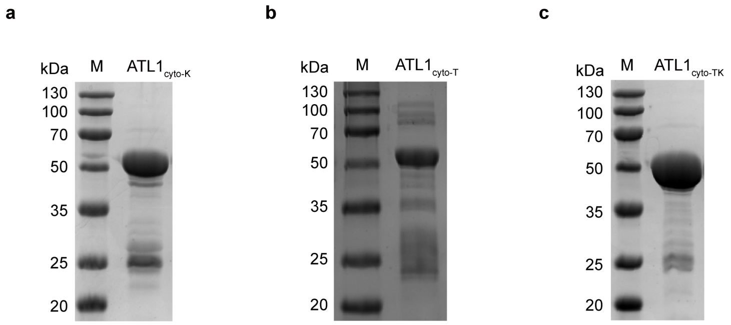

Supplementary Fig. 3 Purities of ATL1$_{\text{cyto-T}}$, ATL1$_{\text{cyto-K}}$, and ATL1$_{\text{cyto-TK}}$. SDS-PAGE of purified ATL1$_{\text{cyto-T}}$ (**a**), ATL1$_{\text{cyto-K}}$ (**b**) and ATL1$_{\text{cyto-TK}}$ (**c**) was analyzed by Coomassie blue staining. M, marker.

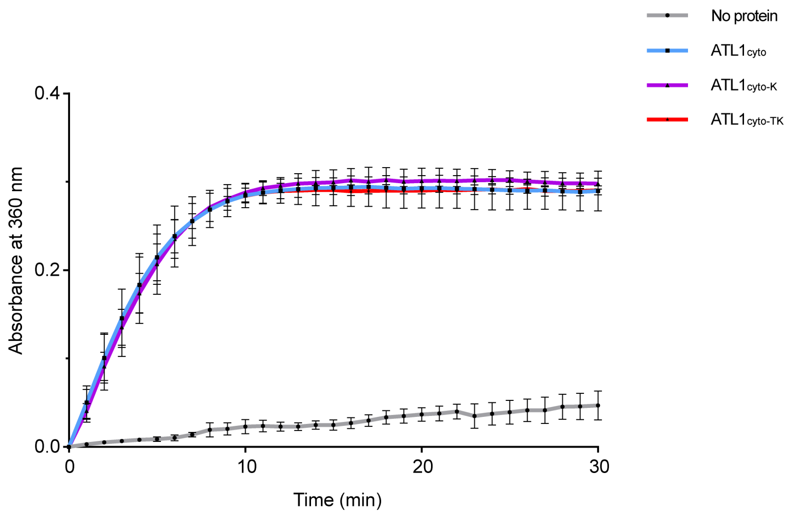

Supplementary Fig. 4 GTPase activities of ATL1$_{cyto}$, ATL1$_{cyto\text{-}K}$, and ATL1$_{cyto\text{-}TK}$. Time courses of the GTPase activities of ATL1$_{cyto}$, ATL1$_{cyto\text{-}K}$ and ATL1$_{cyto\text{-}TK}$ in the presence of GTP as assessed by phosphate release (mean ± SD, n = 3 independent experiments). Source data are provided as a Source Data file.

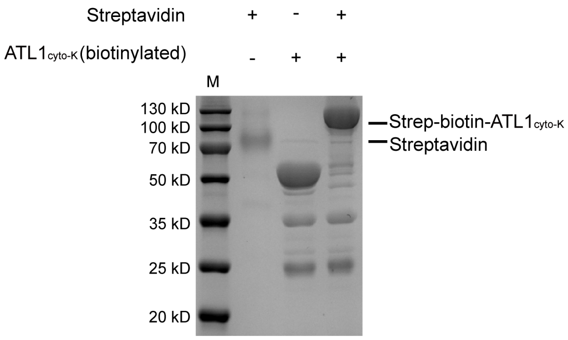

Supplementary Fig. 5 Biotinylation of ATL1$_{\text{cyto-K}}$. Streptavidin, biotinylated ATL$_{\text{cyto-K}}$, and a mix of Streptavidin and biotinylated ATL$_{\text{cyto-K}}$ were separated by SDS-PAGE and analyzed by Coomassie blue staining. M, marker.

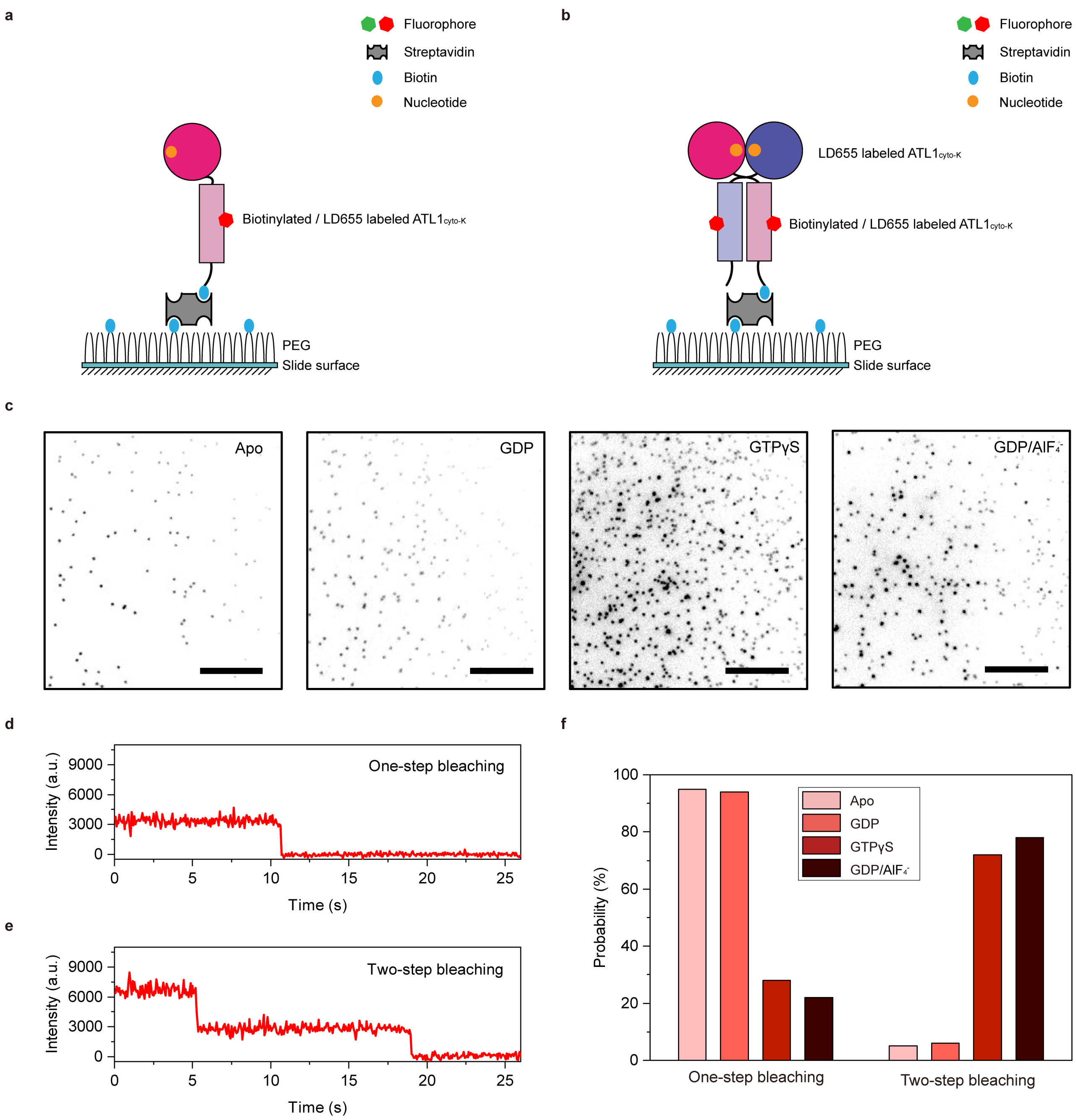

Supplementary Fig. 6 Dimerization of ATL1$_{\text{cyto-K}}$ molecules at the single-molecule level. a-b, Strategy of the single-molecule photobleaching assays for ATL1$_{\text{cyto-K}}$ monomers (**a**) and dimers (**b**) in the presence of different nucleotides. **c,** The immobilized LD655-ATL1$_{\text{cyto-K}}$ molecules in the absence or presence of indicated nucleotide were visualized using a TIRF microscope. Scale bar, 10 µm. **d-e,** Representative photobleaching trajectories of ATL1$_{\text{cyto-K}}$ monomers (**d**) and dimers (**e**). **f,** The relative occupancy of the one-step photobleaching and two-step photobleaching for ATL1$_{\text{cyto-K}}$ in the presence of different nucleotides. Source data are provided as a Source Data file.

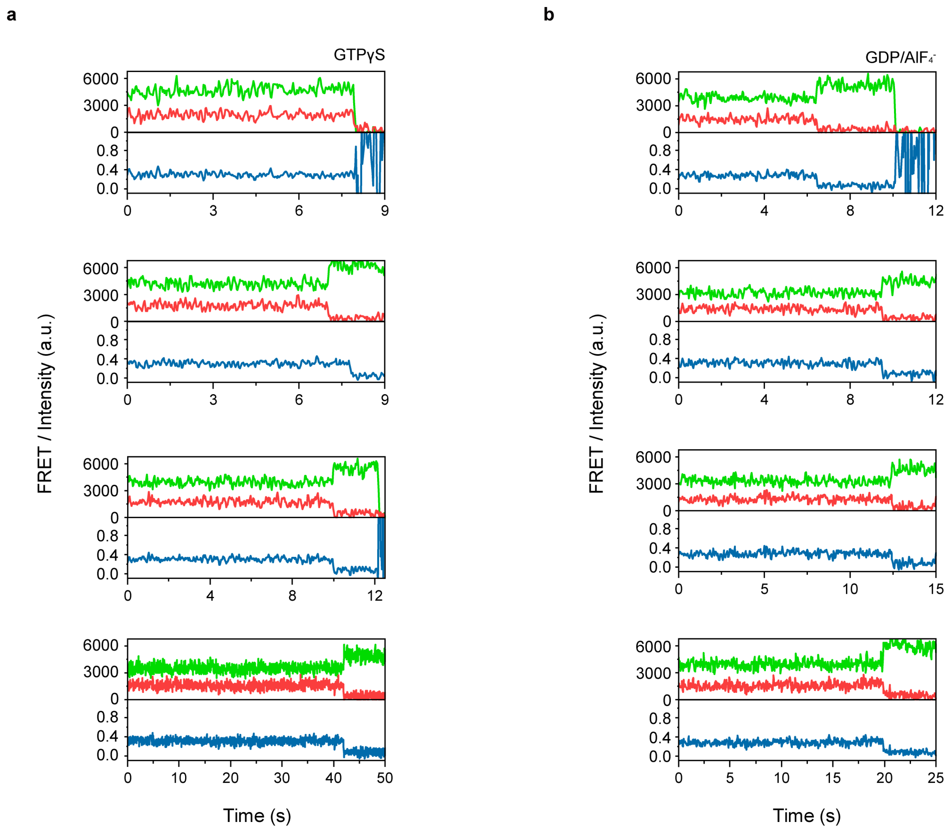

Supplementary Fig. 7 Additional representative fluorescence and smFRET trajectories of intramolecular smFRET assays for ATL1$_{cyto\text{-}K}$-ATL1$_{cyto\text{-}TK}$ dimers in the presence of GTPγS (left) and GDP/AlF_4^- (right). The colors are shown as in Fig. 1c.

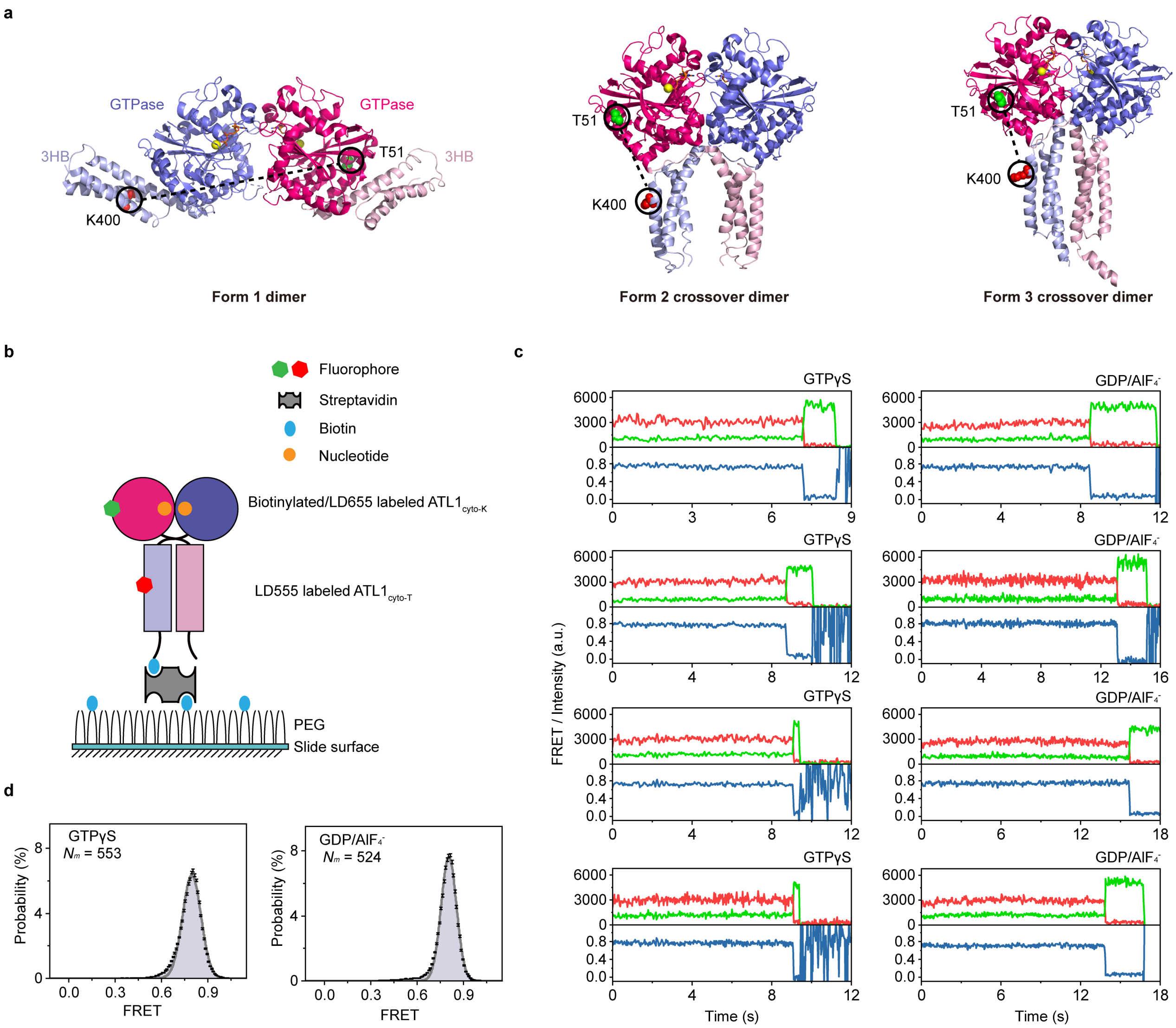

Supplementary Fig. 8 Conformations of ATL1$_{\text{cyto-T}}$-ATL1$_{\text{cyto-K}}$ dimers in the presence of GTPγS and GDP/AlF$_4^-$ revealed by intermolecular smFRET assays. a, As in Fig. 1a, but T51 in one protomer and K400 in the other were selected for dye labeling and are represented as green and red spheres, respectively. **b,** Strategy of the intermolecular smFRET assays for ATL1$_{\text{cyto-T}}$-ATL1$_{\text{cyto-K}}$ dimers in the presence of GTPγS and GDP/AlF$_4^-$. The dimer is formed by an LD555-labeled ATL1$_{\text{cyto-T}}$ and biotinylated LD655-labeled ATL1$_{\text{cyto-K}}$. The biotin-streptavidin interaction was used to immobilize the protein in a streptavidin-coated microfluidic chamber. **c,** Representative fluorescence and smFRET trajectories of intermolecular smFRET assays for ATL1$_{\text{cyto-T}}$-ATL1$_{\text{cyto-K}}$ dimers in the presence of GTPγS (left) and GDP/AlF$_4^-$ (right). LD555 (the donor) is shown in green, LD655 (the acceptor) in red, and FRET in dark blue. **d,** Intermolecular smFRET distributions from ATL1$_{\text{cyto-T}}$-ATL1$_{\text{cyto-K}}$ dimers in the presence of GTPγS (left) and

GDP/AlF_4^- (right). All of the individual FRET values, with the number of molecules (Nm) displayed, were compiled into a conformation-population FRET histogram (gray lines) and fitted into a one-state GaussAmp distribution (~0.80). Each bar height represents the normalized count (%). The length of the error bar represents the normalized SD of a Poissom distribution from the count. Source data are provided as a Source Data file.

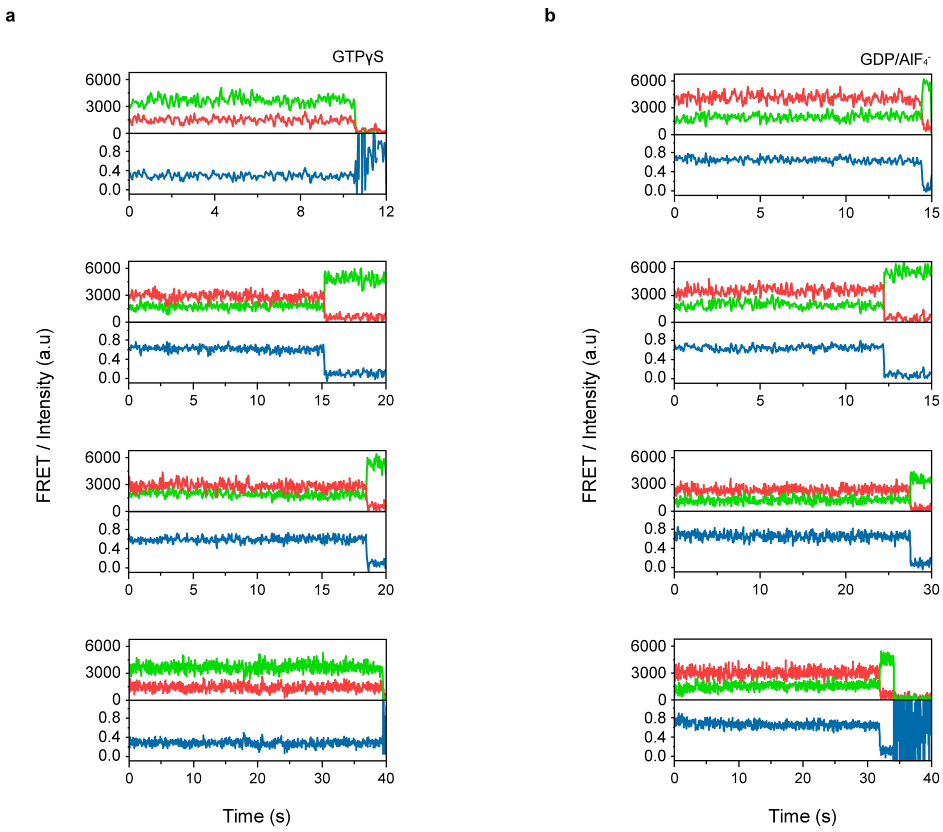

Supplementary Fig. 9 Additional representative fluorescence and smFRET trajectories of intermolecular smFRET assays for ATL1$_{cyto-K}$ dimers in the presence of GTPγS (left) and GDP/AlF_4^- (right). The colors are shown as in Fig. 2c.

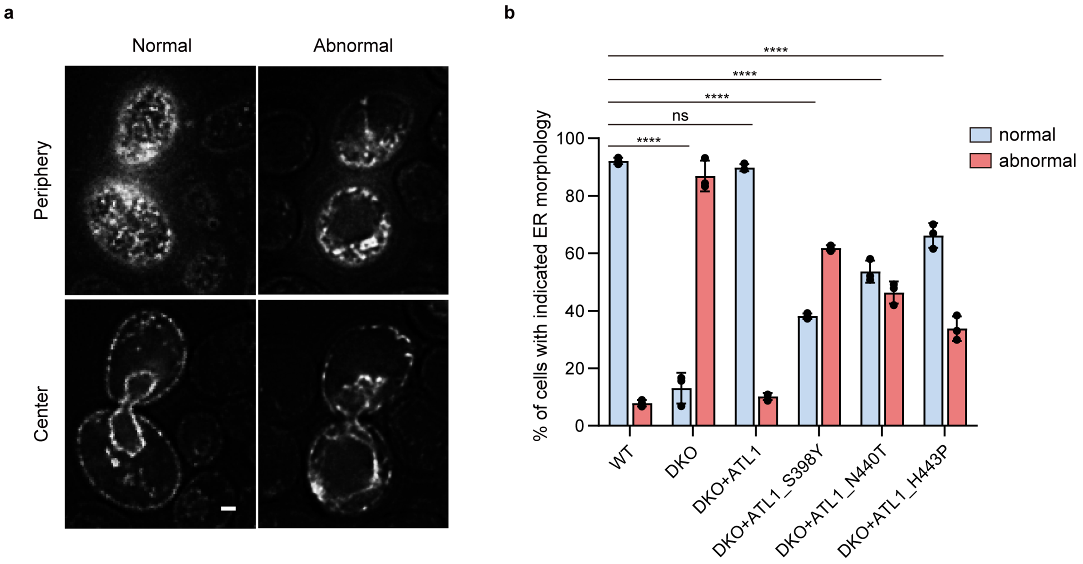

Supplementary Fig. 10 Function of ATL1 analyzed *in vivo* in yeast cells. a, The ER morphology in yeast cells was visualized by expressing ER membrane protein Sec63p with a GFP tag. Either the periphery or the center of the cells was focused. The ER morphology at the periphery of the cell was categorized into two classes. Scale bar, 1 µm. **b,** The ER morphology at the periphery of the WT yeast cells or the *sey1Δyop1Δ* yeast cells (DKO) expressing the indicated ATL1 protein under the endogenous SEY1 promoter was analyzed. Data are presented as mean ± SD (n = 479, 405, 373, 364, 422, and 369 cells from left to right). ns, not significant; **** P<0.0001 by one-way ANOVA with Tukey's multiple comparisons test. Source data are provided as a Source Data file.

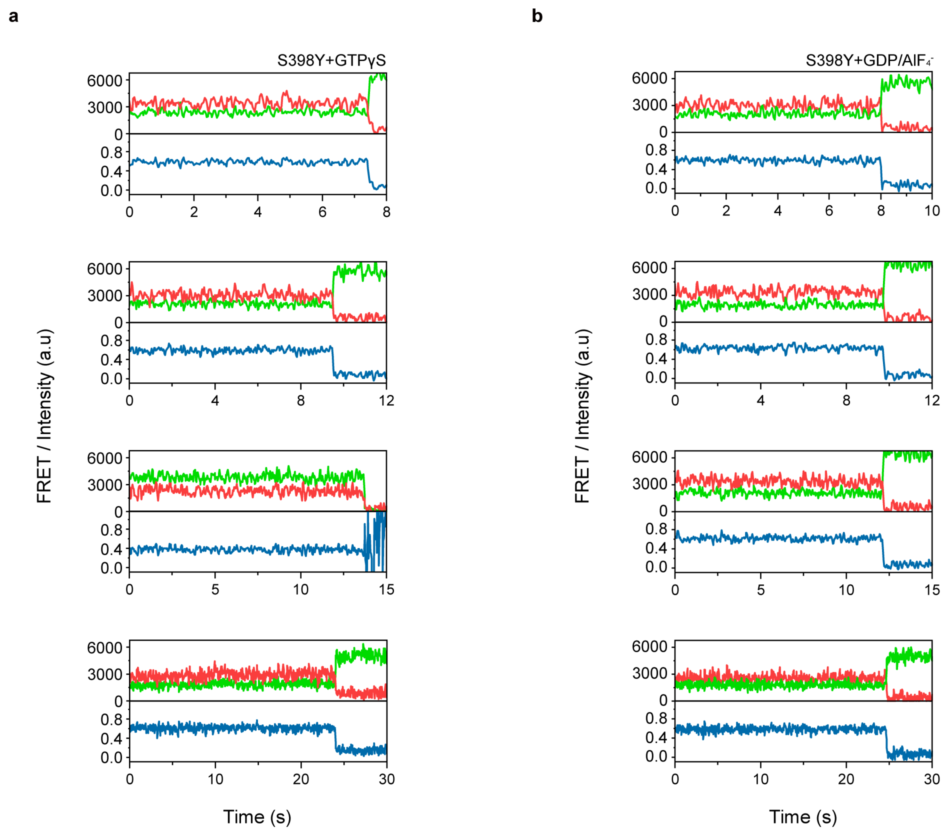

Supplementary Fig. 11 Additional representative fluorescence and smFRET trajectories of intermolecular smFRET assays for ATL1$_{cyto\text{-}K}$-S398Y dimers in the presence of GTPγS (left) and GDP/AlF_4^- (right). The colors are shown as in Fig. 3b.

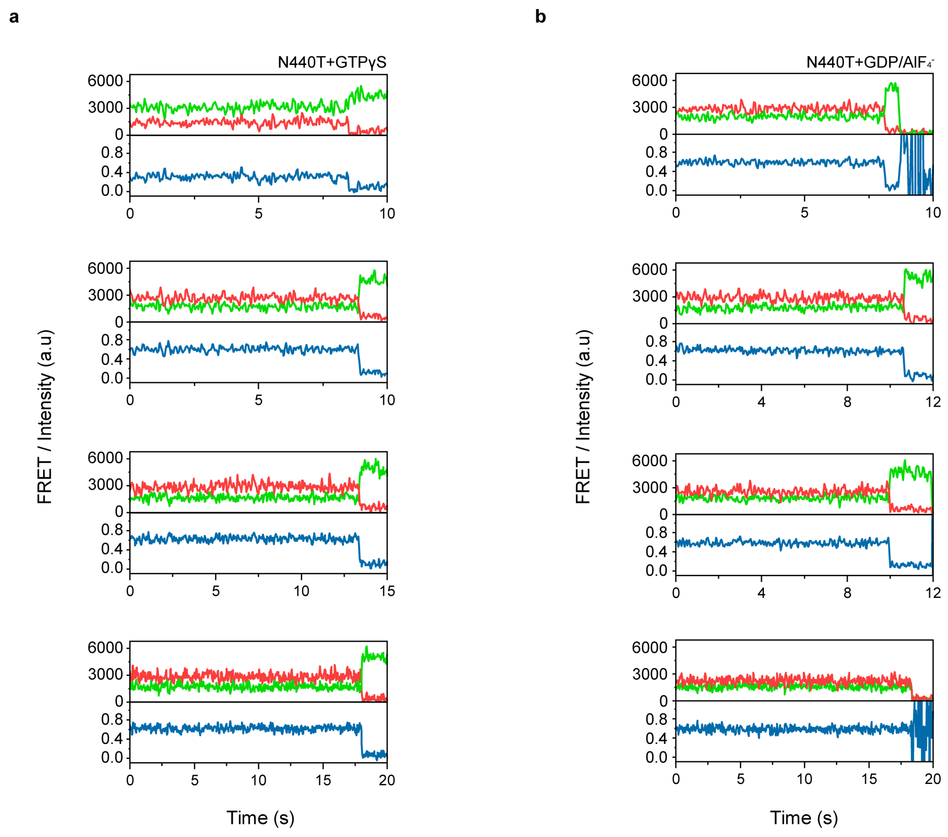

Supplementary Fig. 12 Additional representative fluorescence and smFRET trajectories of intermolecular smFRET assays for ATL1$_{\text{cyto-K}}$-N440T dimers in the presence of GTPγS (left) and GDP/AlF_4^- (right). The colors are shown as in Fig. 3g.

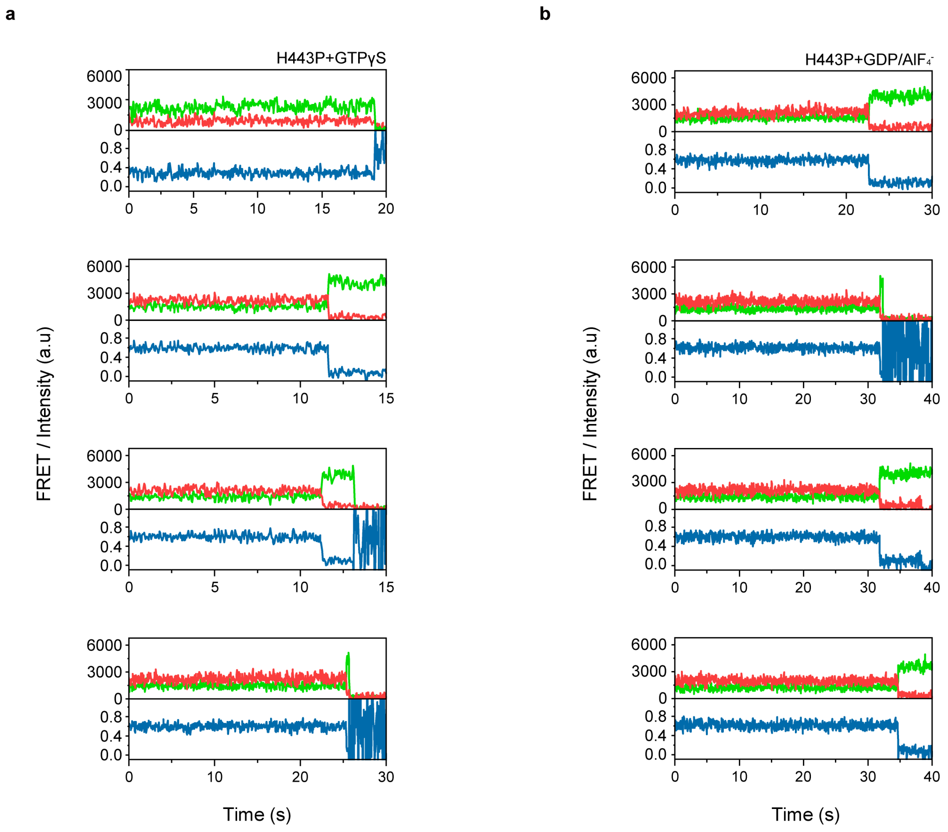

Supplementary Fig. 13 Additional representative fluorescence and smFRET trajectories of intermolecular smFRET assays for ATL1$_{\text{cyto-K}}$-H443P dimers in the presence of GTPγS (left) and GDP/AlF$_4^-$ (right). The colors are shown as in Fig. 3j.

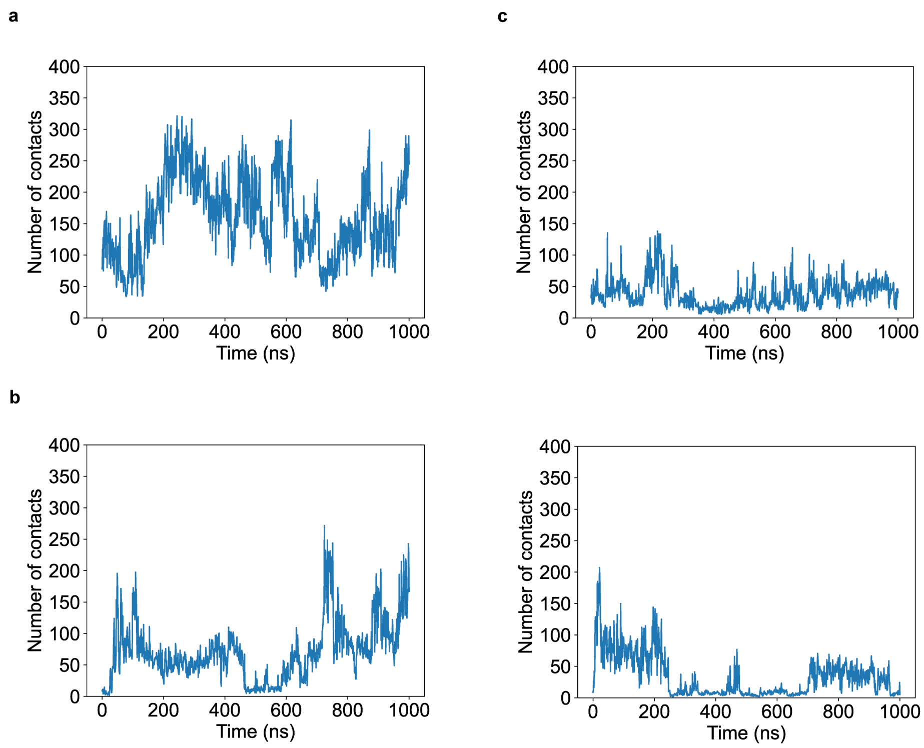

Supplementary Fig. 14 Interaction of the helical motifs linking the 3HBs to the TMDs of ATL1 molecules. a, MD trajectory of the number of contacts between the helical motifs in Form 3 dimer. This is the repeated experiment result for Fig. 4e. **b,** As in (**a**), but in Form 2 dimer. These are two repeat experiment results. **c,** As in (**a**), but with ATL1-N440T. These is the repeat experiment result for Fig. 4g.

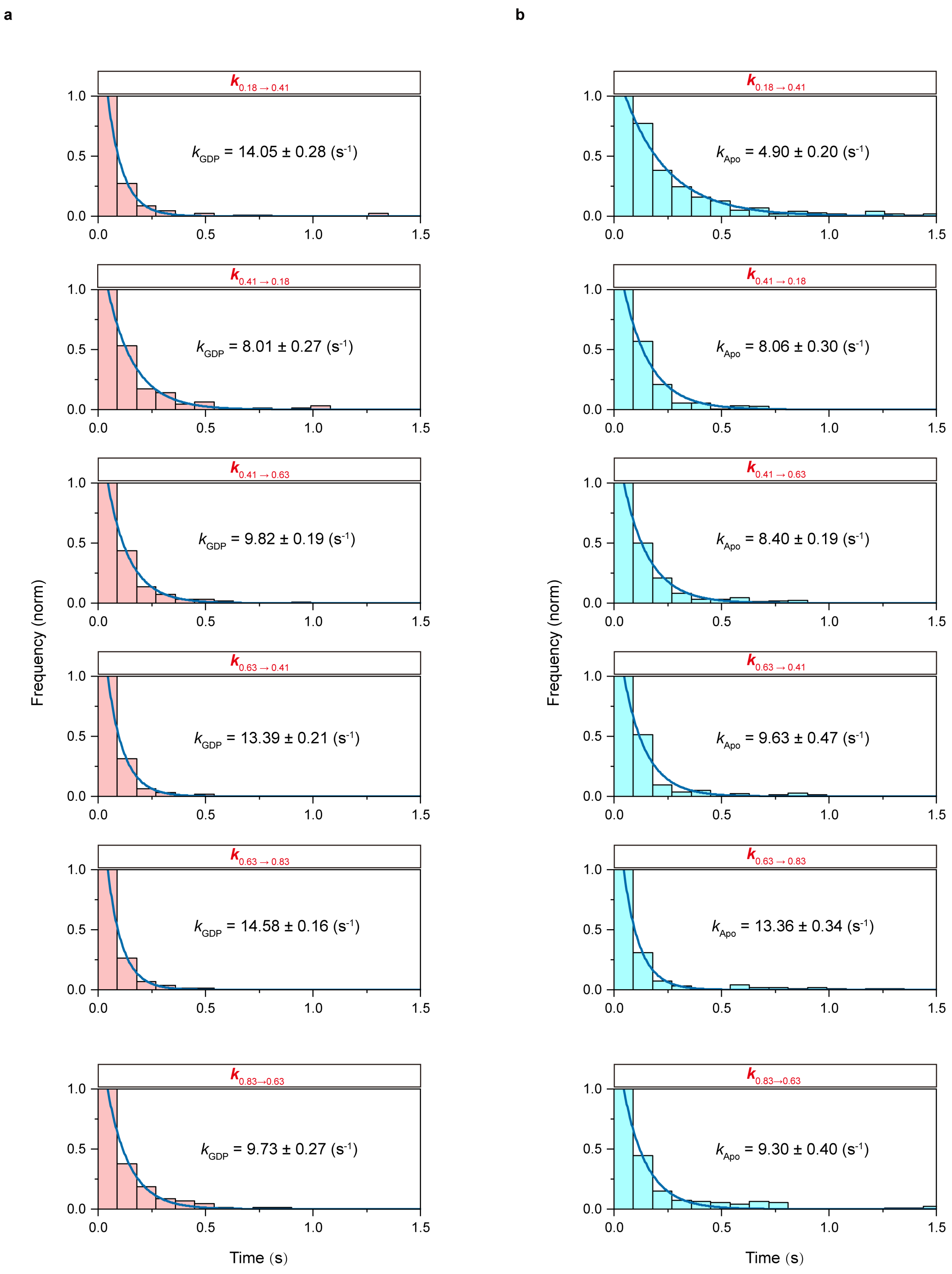

Supplementary Fig. 15 Dwell time distributions of ATL1$_{cyto\text{-}TK}$ monomers (related to Fig. 5). Distributions were plotted as the duration in each FRET state in the absence (right) or presence of GDP (left) before state-to-state transitions occur. Transition rate constants were calculated by

single exponential fitting of duration dwelling on conformations before state transition. Data are presented as mean ± SEM. Source data are provided as a Source Data file.

Supplementary Table 1. Fitting parameters for intramolecular FRET of $ATL1_{cyto\text{-}TK}$ in the presence of GTPγS or GDP/AlF_4^-.

Experimental Conditions	Curve Fitting R-squared	FRET Fitting Central Value	Dye-dye Distance
		μ: 0.28 ± 0.03	R_0: 62 Å
		σ: 0.10 ± 0.01	
+ GTPγS	0.99878	0.28207	72 Å
+ GDP/AlF_4^-	0.99882	0.29236	72 Å

Supplementary Table 2. The Cα-Cα and theoretical dye-dye distances.

	Form 1	Form 2	Form 3
**Intramolecular distance**			
$ATL1_{cyto\text{-}TK}$ Cα-Cα distance	31 Å	64 Å	58 Å
$ATL1_{cyto\text{-}TK}$ theoretical dye-dye distance	49 ± 6 Å	86 ± 5 Å	82 ± 4 Å
**Intermolecular distance**			
$ATL1_{cyto\text{-}T}$-$ATL1_{cyto\text{-}K}$ dimer Cα-Cα distance	88 Å	42 Å	41 Å
$ATL1_{cyto\text{-}T}$-$ATL1_{cyto\text{-}K}$ dimer theoretical dye-dye distance	Not applied	51 ± 4 Å	50 ± 4 Å
$ATL1_{cyto\text{-}K}$-$ATL1_{cyto\text{-}K}$ dimer Cα-Cα distance	106 Å	43 Å	34 Å
$ATL1_{cyto\text{-}K}$-$ATL1_{cyto\text{-}K}$ dimer theoretical dye-dye distance	Not applied	65 ± 2 Å	53 ± 3 Å

The theoretical dye-dye distances were calculated using FRETpredict (https://pypi.org/project/FRETpredict/).

Supplementary Table 3. Fitting parameters for intermolecular FRET of $ATL1_{cyto-T}$-$ATL1_{cyto-K}$ dimer in the presence of GTPγS or GDP/AlF_4^-.

Experimental Conditions	Curve Fitting R-squared	FRET Fitting Central Value	Dye-dye Distance
		μ: 0.80 ± 0.01	R_0: 62 Å
		σ: 0.05 ± 0.01	
+ GTPγS	0.99968	0.79588	49 Å
+ GDP/AlF_4^-	0.99595	0.80461	49 Å

Supplementary Table 4. Fitting parameters for intermolecular FRET of ATL1$_{cyto-K}$ and its mutations in the presence of GTPγS or GDP/AlF_4^-.

Experimental Conditions	Curve Fitting R-squared	Low-FRET Fitting central value	Dye-dye Distance	High-FRET Fitting central value	Dye-dye Distance
		μ: 0.28 ± 0.03	R_0: 62 Å	μ: 0.66 ± 0.02	R_0: 62 Å
		σ: 0.14 ± 0.02		σ: 0.13 ± 0.02	
WT + GTPγS	0.99777	0.25716	74 Å	0.67720	55 Å
WT + GDP/AlF_4^-	0.99808			0.67809	55 Å
S398Y + GTPγS	0.99879	0.25584	74 Å	0.64043	56 Å
S398Y + GDP/AlF_4^-	0.99859			0.66006	56 Å
N440T + GTPγS	0.99795	0.25000	74 Å	0.65723	56 Å
N440T + GDP/AlF_4^-	0.99887			0.68000	55 Å
H443P + GTPγS	0.99933	0.31000	71 Å	0.64062	56 Å
H443P + GDP/AlF_4^-	0.99854			0.68000	55 Å

Supplementary Table 5. Summary of MD simulations.

Protein	Ligand	State	Simulation Time
ATL1	-	Monomer	1000 ns
ATL1	GDP/Pi	Form 3 dimer	1000 ns
ATL1-N440T	GDP/Pi	Form 3 dimer	1000 ns
ATL1	GDP	Form 2 dimer	1000 ns

Supplementary Table 6. Fitting parameters for intramolecular FRET of ATL1$_{cyto\text{-}TK}$ in the absence or presence of GDP.

**Experimental conditions**	**Curve fitting R-squared**	**Ultra-low-FRET Fitting Central Value**	**Dye-dye Distance**	**Medium-FRET Fitting Central Value**	**Dye-dye Distance**	**High-FRET Fitting Central Value**	**Dye-dye Distance**	**Ultra-high-FRET Fitting Central Value**	**Dye-dye Distance**
		μ: 0.18 ± 0.01	R_0: 62 Å	μ: 0.41 ± 0.01	R_0: 62 Å	μ: 0.63 ± 0.01	R_0: 62 Å	μ: 0.83 ± 0.01	R_0: 62 Å
		σ: 0.11 ± 0.02		σ: 0.11 ± 0.02		σ: 0.11 ± 0.02		σ: 0.11 ± 0.02	
Apo	0.99856	0.18022	80 Å	0.40033	66 Å	0.62000	57 Å	0.82000	48 Å
+ GDP	0.99893	0.18802	79 Å	0.41313	66 Å	0.62000	57 Å	0.82540	48 Å

Supplementary Table 7. Correlation coefficient γ in intramolecular and intermolecular smFRET experiments under different nucleotide conditions.

	Apo	GTPγS	GDP/AlF_4^-	GDP
**Intramolecular smFRET**				
$ATL1_{cyto-TK}$	0.76	0.84	0.72	0.83
**Intermolecular smFRET**				
$ATL1_{cyto-T}$-$ATL1_{cyto-K}$ dimer	-	0.78	0.78	-
$ATL1_{cyto-K}$ dimer	-	0.71	0.82	-
$ATL1_{cyto-K}$-S398Y dimer	-	0.75	0.70	-
$ATL1_{cyto-K}$-N440T dimer	-	0.74	0.65	-
$ATL1_{cyto-K}$-H443P dimer	-	0.69	0.73	-
