# Supplementary material for: Dissecting the mechanism of atlastin-mediated homotypic membrane fusion at the single-molecule level
Source: Nat Commun. 2024 Mar 20;15:2488. doi: 10.1038/s41467-024-46919-z (PMC10954664; doi:10.1038/s41467-024-46919-z)
Supplement: Supplementary file 3 — Description of Additional Supplementary Files [file 41467_2024_46919_MOESM3_ESM.pdf]

Description of Additional Supplementary Files

File Name: Supplementary Movie 1

Description: A MD simulation of the GDP/Pi-bound Form 3 ATL1 crossover dimer within a POPC lipid bilayer. The simulation starts from the AlphaFold2 predicted structure of the ATL1 dimer. In the cartoon representation, the helical regions and transmembrane domains of the ATL1 dimer are colored in green and light blue, respectively. The phosphate head groups of the POPC lipids are shown as white spheres.
